# The Application of Machine Learning in Predicting the Permeability of Drugs Across the Blood Brain Barrier

**DOI:** 10.5812/ijpr-149367

**Published:** 2024-11-24

**Authors:** Sogand Jafarpour, Maryam Asefzadeh, Ehsan Aboutaleb

**Affiliations:** 1 School of Pharmacy, Guilan University of Medical Sciences, Rasht, Iran; 2 Department of Pharmaceutics, School of Pharmacy, Guilan University of Medical Sciences, Rasht, Iran

**Keywords:** Blood-Brain Barrier, Permeability, B3DB, Machine Learning

## Abstract

The inefficiency of some medications to cross the blood-brain barrier (BBB) is often attributed to their poor physicochemical or pharmacokinetic properties. Recent studies have demonstrated promising outcomes using machine learning algorithms to predict drug permeability across the BBB. In light of these findings, our study was conducted to explore the potential of machine learning in predicting the permeability of drugs across the BBB.

We utilized the B3DB dataset, a comprehensive BBB permeability molecular database, to build machine learning models. The dataset comprises 7,807 molecules, including information on their permeability, stereochemistry, and physicochemical properties. After preprocessing and cleaning, various machine learning algorithms were implemented using the Python library Pycaret to predict permeability.

The extra trees classifier model outperformed others when using Morgan fingerprints and Mordred chemical descriptors (MCDs), achieving an area under the curve (AUC) of 0.93 and 0.95 on the test dataset. Additionally, we conducted an experiment to train a voting classifier combining the top three performing models. The best-blended model, trained on MCDs, achieved an AUC of 0.96. Furthermore, Shapley additive exPlanations (SHAP) analysis was applied to our best-performing single model, the extra trees classifier trained on MCDs, identifying the Lipinski rule of five as the most significant feature in predicting BBB permeability.

In conclusion, our combined model trained on MCDs achieved an AUC of 0.96, an F1 Score of 0.91, and an MCC of 0.74. These results are consistent with prior studies on CNS drug permeability, highlighting the potential of machine learning in this domain.

## 1. Background

The central nervous system (CNS) is separated from the bloodstream by the blood-brain barrier (BBB), a highly selective barrier primarily formed by the endothelium of brain capillaries. The BBB prevents many molecules from entering the CNS, allowing only selective transporters and certain water- and lipid-soluble molecules to pass through. Active efflux systems, such as P-glycoprotein (Pgp), are present in the BBB to block neurotoxins, but they also impede the entry of some drugs (1). The inefficiency of certain drugs in crossing the BBB is often due to their poor physicochemical or pharmacokinetic properties, such as inefficient absorption, distribution, metabolism, and excretion (ADME) (2). The pharmacokinetics of a drug in the plasma can differ significantly from its pharmacokinetics in the brain. Studying CNS-specific drug pharmacokinetics requires understanding the relationship between the drug’s physicochemical properties and the physiological functions of the BBB (3). In recent years, with the development of artificial intelligence, various statistical methods and machine learning algorithms have been utilized to make such predictions. Machine learning, a subset of artificial intelligence, aims to enable computer programs to automatically learn patterns within data (4).

Recent advancements in artificial intelligence have facilitated the use of statistical methods and machine learning algorithms to predict BBB permeability. Machine learning, a subset of artificial intelligence, focuses on developing algorithms and statistical models that enable computer systems to learn patterns within data and improve their performance without explicit programming (4, 5). Techniques such as logistic regression (6), support vector machines (SVMs) (7), and K-nearest neighbors (KNNs) (8) are commonly used to identify patterns and relationships in data. While these algorithms have inherent strengths and weaknesses, their performance can be enhanced by fine-tuning hyperparameters (9). For example, adjusting hyperparameters like learning rate and regularization strength can optimize the algorithm’s performance and improve prediction accuracy (10).

These machine learning approaches are not limited to predicting drug permeability to the BBB. They are also employed in fields such as predicting protein folding to determine a protein’s three-dimensional structure, protein-protein interactions, and ligand-based virtual screening when detailed information about a protein's structure is unavailable (11).

Recent studies have highlighted the growing interest in using machine learning for predicting drug properties. Saber et al. compared sequential feature selection and genetic algorithms to predict BBB permeability (12). Shaker et al. used a large dataset of 7,162 compounds to train a machine learning model based on the light gradient boosting machine (light GBM) algorithm for BBB permeability prediction (13). Building on these findings, our study aimed to further investigate the potential of machine learning in predicting drug permeability to the BBB, focusing on optimizing algorithms and fine-tuning hyperparameters for greater prediction accuracy.

We conducted a comprehensive analysis of various machine learning techniques, including logistic regression, SVMs, and KNNs. Additionally, we integrated advancements such as the light GBM algorithm, which has demonstrated promising results in prior studies. Beyond merely predicting BBB permeability, a significant part of our research focused on interpreting the results and identifying critical descriptors.

Interpretability is crucial for understanding the decision-making process of machine learning models. By analyzing feature importance, we identified key descriptors that significantly influence prediction outcomes. These descriptors often represent specific physicochemical or pharmacokinetic properties of drug compounds. Understanding their importance validates the model’s predictive capabilities and provides essential insights for drug design and development. By focusing on optimizing these descriptors, researchers can design drugs with improved BBB permeability. This interpretability bridges the gap between complex algorithmic predictions and practical pharmacokinetic applications, enhancing the utility of machine learning in drug development.

## 2. Methods

### 2.1. Data Collection

We developed and evaluated machine learning models to predict the BBB permeability of drug-like molecules using the B3DB dataset, a curated molecular database focused on BBB permeability with comprehensive chemical descriptors. This database, compiled from 50 published sources, represents the largest collection of numerical and categorical data for small molecules associated with BBB permeability. It includes 7,807 molecules, of which 4,956 are classified as permeable and 2,851 as non-permeable to the BBB.

The dataset provides detailed information, including stereochemistry, chiral characteristics, and molecular representations using the simplified molecular input line entry system (SMILES), a linear notation method for describing molecules and chemical reactions. Additionally, pre-calculated physicochemical features generated through the Mordred library are included in the dataset (14).

### 2.2. Preprocessing

We employed supervised learning to train artificial intelligence models, with the reference label being the presence or absence of a molecule’s permeability to the BBB, as extracted from the B3DB library. The input data for the models consisted of the structural and physicochemical information of molecules represented using SMILES strings (15) available in the B3DB library. After extracting SMILES information and molecule labels, we utilized the RDKit (16) and Mordred (17) libraries to derive numerical features for each molecule. These features included extended-connectivity fingerprints with a diameter of six (ECFP6), which numerically describe molecular activity (18). Morgan fingerprints, also known as extended connectivity fingerprints (19), and Mordred chemical descriptors (MCDs) (17) were also employed. Mordred chemical descriptors provide numerical values corresponding to two-dimensional and three-dimensional descriptors of a molecule.

We converted ECFP6 and Morgan fingerprints into bit representations for machine learning calculations. Both fingerprints comprised 2,048 bits to represent each molecule, differing only in their computational methods. Using the Mordred library, we extracted 1,826 descriptors for each molecule. These chemical descriptors contained numerical and non-numerical information for each molecule. Since not all descriptors were available for every molecule, preprocessing was necessary. We cleaned the dataset by removing descriptors with non-numerical information, descriptors missing values for more than 30 molecules, columns with constant values, and descriptors with a standard deviation to mean ratio of less than 0.05. After this cleaning step, the dataset retained 7,775 molecules with 912 descriptors each.

Following the initial cleaning, we evaluated the relationship between each descriptor and others to eliminate redundancy. For any two highly correlated descriptors, we retained only one to avoid redundant information entering the model. A Pearson correlation coefficient threshold of 0.95 was used to identify highly correlated descriptors. After this second cleaning phase, the dataset comprised 7,763 molecules with 393 descriptors.

### 2.3. Modeling

We employed the open-source Python low-code machine learning library named PyCaret for model development. Leveraging its low-code functionality, PyCaret simplifies machine learning workflows by enabling efficient model management on the Python platform. PyCaret integrates various machine-learning libraries and frameworks, including scikit-learn, XGBoost, LightGBM, CatBoost, spaCy, Optuna, Hyperopt, and Ray (20). PyCaret features hyperparameter tuning, which helps identify optimal hyperparameters to prevent overfitting, and early stopping, which halts the training process when the model’s performance on the validation set begins to degrade, thereby avoiding overfitting (21).

We evaluated and trained all available models within this library. These models include light GBM, gradient boosting machine (GBM), AdaBoost, Random Forest (RF), Decision Tree (DT), extra trees classifier, KNN, linear discriminant analysis (LDA), Ridge Classifier, quadratic discriminant analysis (QDA), naive Bayes (NB), Support Vector Machine (SVM), and Logistic Regression (LR). Light GBM is a gradient-boosting framework that employs tree-based learning algorithms and is renowned for its efficiency and scalability with large datasets (22). Gradient boosting machine is a robust algorithm that constructs an ensemble of weak prediction models and integrates them to create a stronger, more effective model capable of handling complex datasets (23). AdaBoost, on the other hand, combines multiple weak learners to form a strong learner. This algorithm trains the AdaBoost model by adjusting the training set based on the accuracy of the previous iteration's predictions. It assigns greater weight to misclassified observations, ensuring they receive higher classification probabilities in subsequent iterations (24). Random forest is an ensemble learning algorithm that combines multiple decision trees to make predictions. It is easy to use and can handle both regression and classification problems (25). Decision tree is a straightforward tree-based algorithm that creates a model by splitting the data into smaller subsets based on the value of a single feature. It can manage both categorical and numerical data (26). Extra trees classifier, another ensemble algorithm, builds multiple decision trees and uses them to predict outcomes. Unlike RF, it randomly selects features to split on instead of searching for the best feature (27). K-nearest neighbor bases its predictions on the (k) closest data points in the training set (28). Linear discriminant analysis is a statistical algorithm that seeks a linear combination of features that best separates the classes in the data, making it particularly useful in classification problems (29). Ridge classifier is a linear algorithm that employs L2 regularization to prevent overfitting (30). Quadratic discriminant analysis is similar to LDA but allows for non-linear separation between classes (31).

Naive Bayes methods utilize supervised learning algorithms based on Bayes' theorem. The "naive" assumption is that every pair of features is conditionally independent given the value of the class variable (32). Support vector machine is a linear algorithm that tries to find a hyperplane that best separates the classes in the data (33). Lastly, LR is a widely used algorithm for predicting permeability (34).

**Figure 1.**
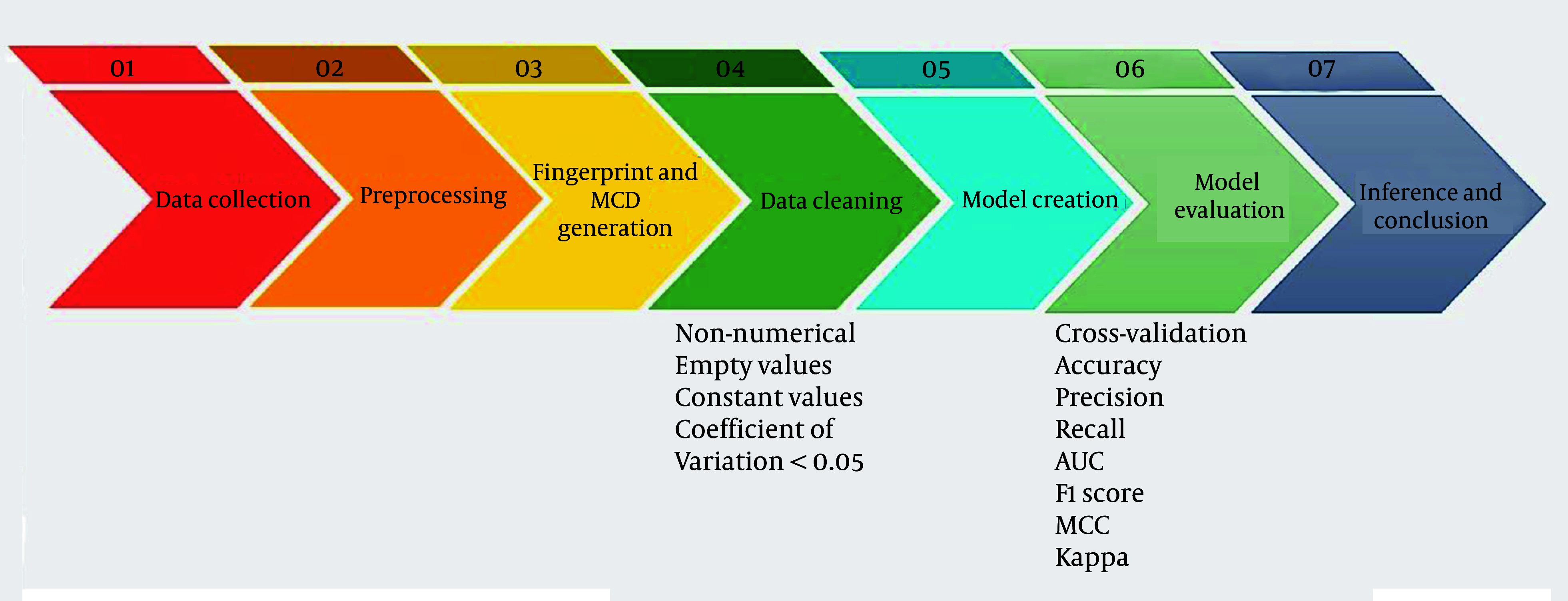
Process flowchart

Beyond individual models, we explored the ensemble voting method. Ensemble voting is a machine learning technique that combines predictions from multiple models to improve accuracy and robustness. This can be implemented using hard voting (majority vote) or soft voting (probability averaging). By leveraging the strengths of diverse models, ensemble voting enhances generalization and reduces errors. It has demonstrated potential in various applications, such as medical diagnostics, by improving predictive performance and decision-making processes (35). Our study aimed to boost the overall predictive performance and robustness of our machine-learning solutions. The flowchart of the process is presented in Figure 1. 

### 2.4. Evaluation

We split the data into two parts for model evaluation: 80% for training (6,210 molecules) and 20% for testing (1,553 molecules). To enhance the efficiency of our algorithm and ensure that the model produces consistent and replicable results, we employed a 10-fold cross-validation technique during the training process. This method divides the training dataset into ten equally sized subsets. Nine subsets are used for training the model, while the remaining one is reserved for testing. This process is repeated ten times, with each subset serving as the testing set once. By adopting this approach, we evaluated the model's performance across various data subsets, which helped to detect potential weaknesses or biases. Ultimately, the data split resulted in 4,347 molecules used for training, 1,863 for validation, and 1,553 as the test set.

We used several performance metrics to assess the proposed algorithms' effectiveness, including accuracy, precision, recall, the area under the curve (AUC), F1-score, Matthew’s correlation coefficient (MCC), and Kappa (36).

To interpret the results of our models, we utilized the Shapley additive exPlanations (SHAP) library. Shapley additive exPlanations is a game theory-based method designed to explain the output of any machine learning model. It links optimal credit allocation with local explanations by leveraging classic Shapley values from game theory and their extensions. Using the SHAP library, we identified the most significant features contributing to the model's predictions, providing valuable insights into the underlying factors driving the results (37).

## 3. Results

**Figure 2.**
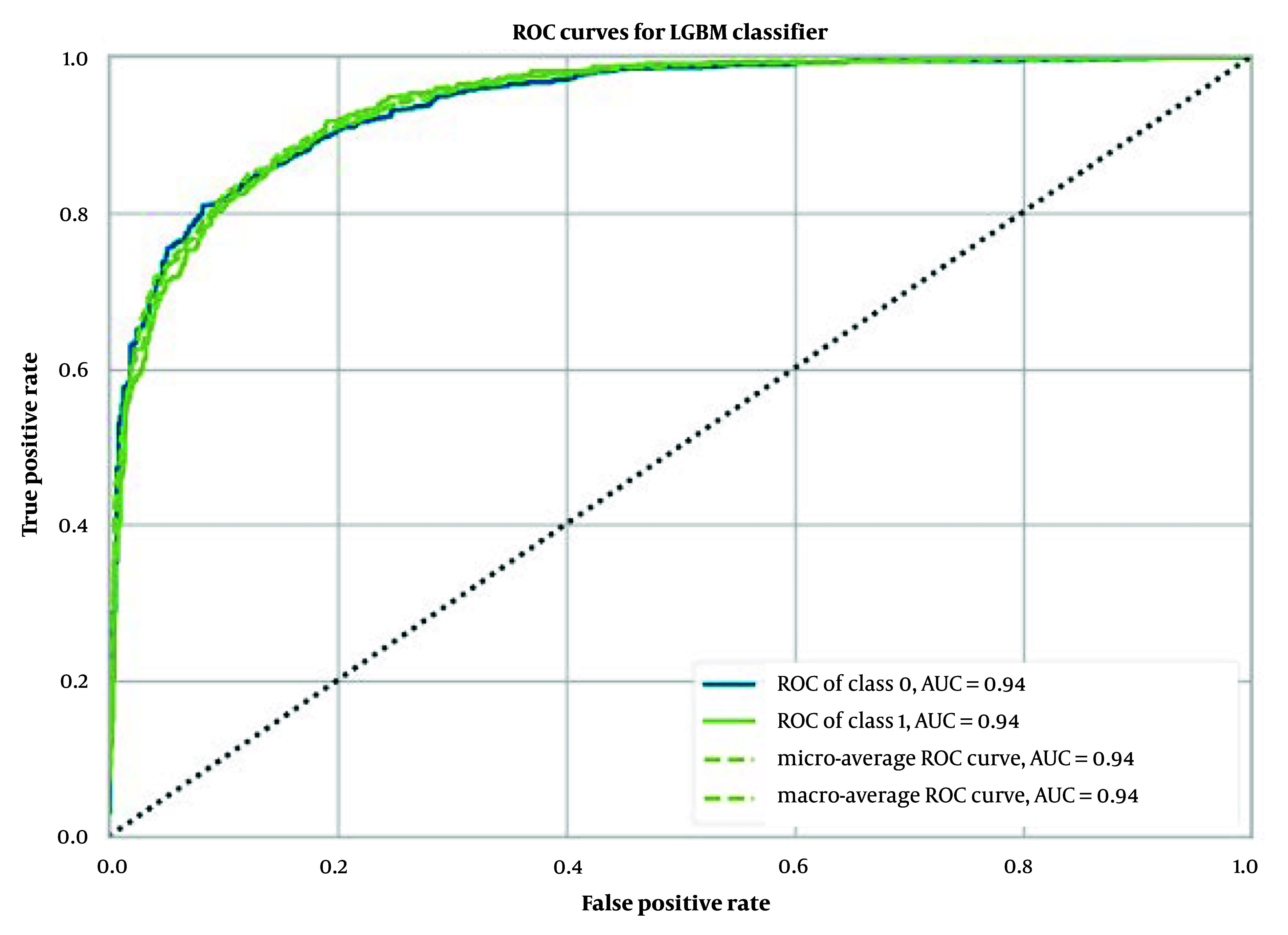
Receiver operating characteristic (ROC) curves for extra trees classifier with ECFP6

In the task of predicting BBB molecular permeability, the Light GBM model using 2048 ECFP6 outperformed other models. The model achieved an average accuracy of 0.87 ± 0.01, an average AUC of 0.94 ± 0.01 (Figure 2), an average Recall of 0.74 ± 0.03, an average Precision of 0.88 ± 0.03, an average F1 score of 0.80 ± 0.02, an average Kappa coefficient of 0.70 ± 0.03, and an average MCC of 0.71 ± 0.03, as shown in Table 1. 

**Table 1.** Comparison of the Best ML Model with 10-fold Cross-Validation for ECFP6, and Morgan Fingerprints, and Mordred Chemical Descriptors ^a^

Features	Best Model	F1 score	MCC ^b^	AUC ^c^
**ECFP6**	Light Gradient Boosting Machine	0.80 ± 0.02	0.71 ± 0.03	0.94 ± 0.01
**Morgan fingerprints**	Extra tree classifier	0.79 ± 0.02	0.71 ± 0.02	0.93 ± 0.01
**MCDs**	Extra tree classifier	0.91 ± 0.01	0.74 ± 0.02	0.94 ± 0.01

Abbreviations: GBM, gradient boosting machine; MCDs, Mordred chemical descriptors; AUC, area under the curve.

^a^ Values are expressed as mean ± SD.

^b^ Matthew’s correlation coefficient.

^c^ the area under the Curve.

**Figure 3.**
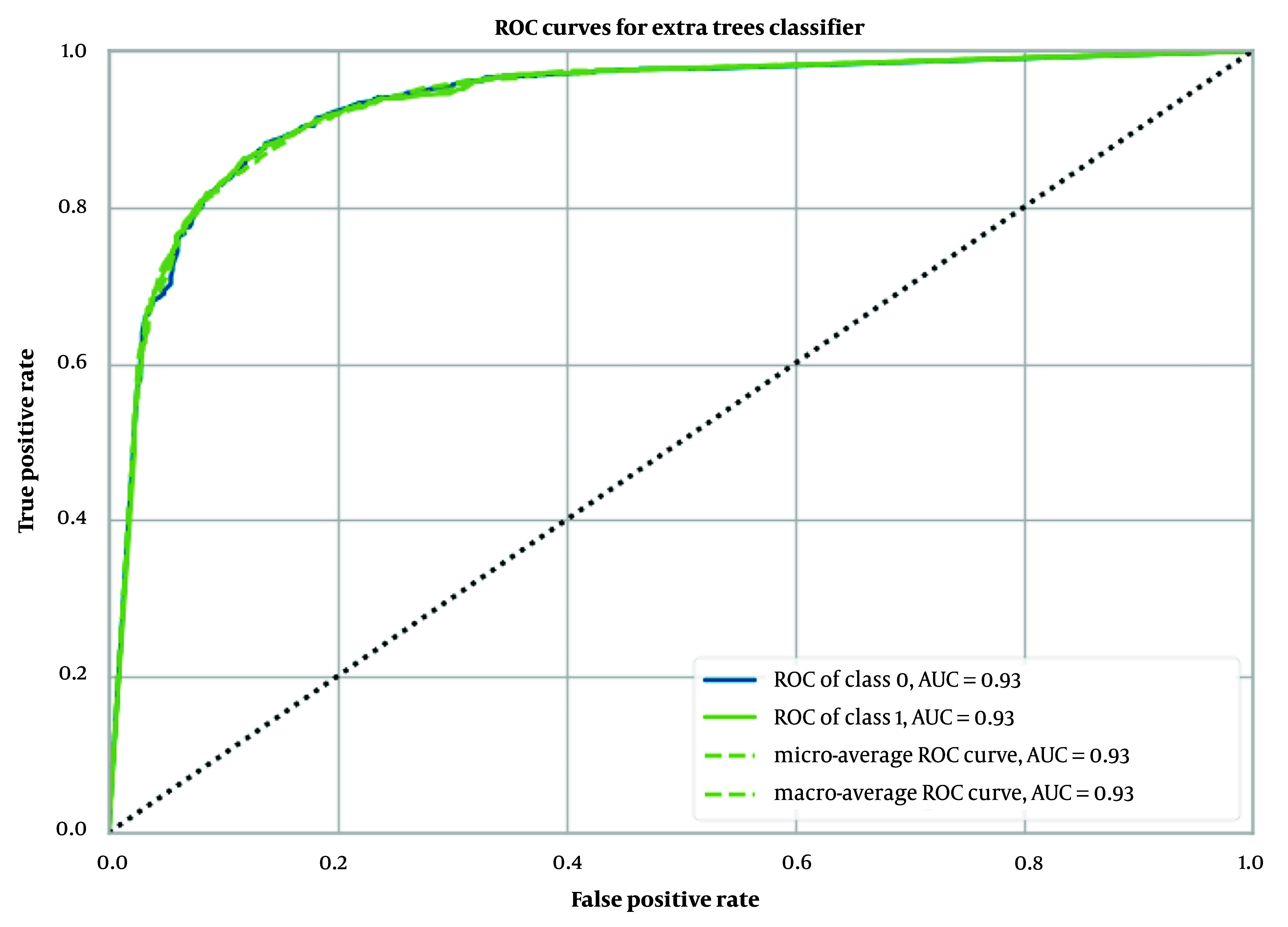
Receiver operating characteristic (ROC) curves for extra trees classifier with Morgan fingerprint

When Morgan fingerprints were used as input, the extra trees classifier model showed superior performance compared to other models. This model achieved an accuracy of 0.87 ± 0.01, an AUC of 0.93 ± 0.01 (Figure 3), a Recall of 0.71 ± 0.03, a Precision of 0.90 ± 0.01, an F1 score of 0.79 ± 0.02, a Kappa coefficient of 0.70 ± 0.02, and an MCC of 0.71 ± 0.02, as outlined in Table 1. 

**Figure 4.**
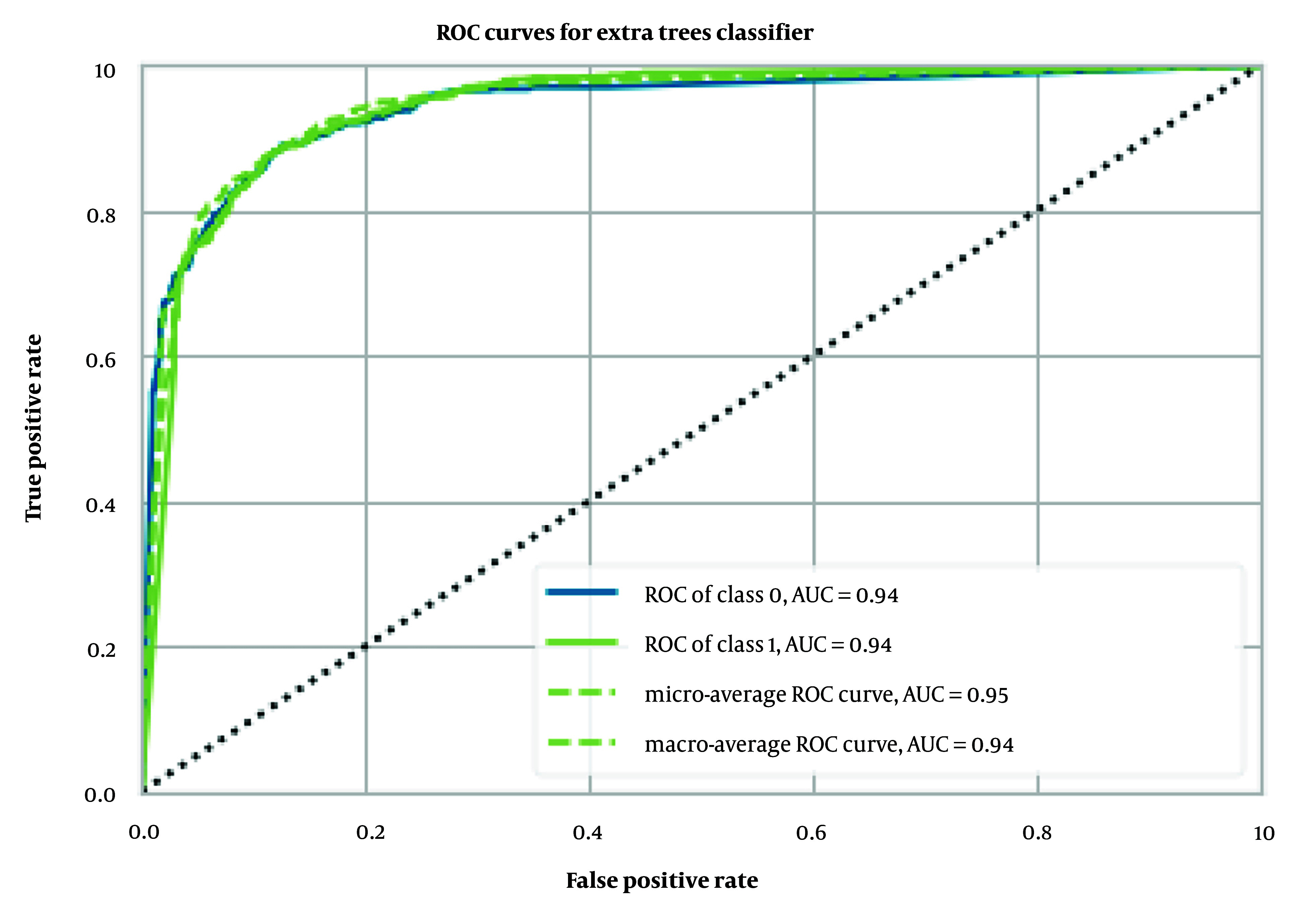
Receiver operating characteristic (ROC) curves for extra trees classifier with Mordred chemical descriptors (MCDs)

Similarly, we implemented and trained the same models using MCDs as input data. Among these models, the extra trees classifier achieved the best results, with an accuracy of 0.88 ± 0.01, an AUC of 0.94 ± 0.01 (Figure 4), a Recall of 0.94 ± 0.01, a Precision of 0.88 ± 0.01, an F1 score of 0.91 ± 0.01, a Kappa coefficient of 0.73 ± 0.02, and an MCC of 0.74 ± 0.02, as detailed in Table 1. 

We also evaluated the performance of the best models on the test dataset, as presented in Table 2. The light GBM trained on ECFP achieved an AUC value of 0.94, an MCC of 0.70, and an F1 score of 0.89. The extra trees classifier trained on Morgan fingerprints achieved an AUC value of 0.93, an MCC of 0.70, and an F1 score of 0.79. The extra trees classifier trained on MCDs achieved an AUC value of 0.95, an MCC of 0.72, and an F1 score of 0.90.

**Table 2.** Comparison of the best ML model for ECFP6, Morgan Fingerprints, and Mordred Chemical Descriptors on the Test Dataset

Features	Model	F1 score	MCC	AUC
**ECFP6**	Light Gradient Boosting Machine	0.89	0.70	0.94
**Morgan Fingerprints**	extra trees classifier	0.79	0.70	0.93
**MCDs**	extra trees classifier	0.90	0.72	0.95

Abbreviation: GBM, gradient boosting machine; MCDs, Mordred chemical descriptors; AUC, area under the curve.

In Table 3, as an additional experiment, we presented the results of the best models combined as a voting classifier. This approach combined the predictions generated by the top three machine learning models to produce the final prediction. The blended model using MCDs demonstrated superior performance on the test dataset compared to the single best model with MCDs. It achieved an AUC of 0.96, an MCC of 0.74, and an F1 score of 0.91.

**Table 3.** Comparison of the Blended ML Model for ECFP6, Morgan Fingerprints, and Mordred Chemical Descriptors on the Test Dataset

Features	Model	F1 score	MCC	AUC
**ECFP6**	Voting Classifier	0.79	0.70	0.86
**Morgan Fingerprint**	Voting Classifier ^a^	0.80	0.72	0.85
**MCDs**	Voting Classifier ^a^	0.91	0.74	0.96

Abbreviations: MCDs, Mordred chemical descriptors; AUC, area under the curve.

^a^ Random Forest classifier + extra trees classifier + light gradient boosting machine, NA, not acceptable results.

**Figure 5.**
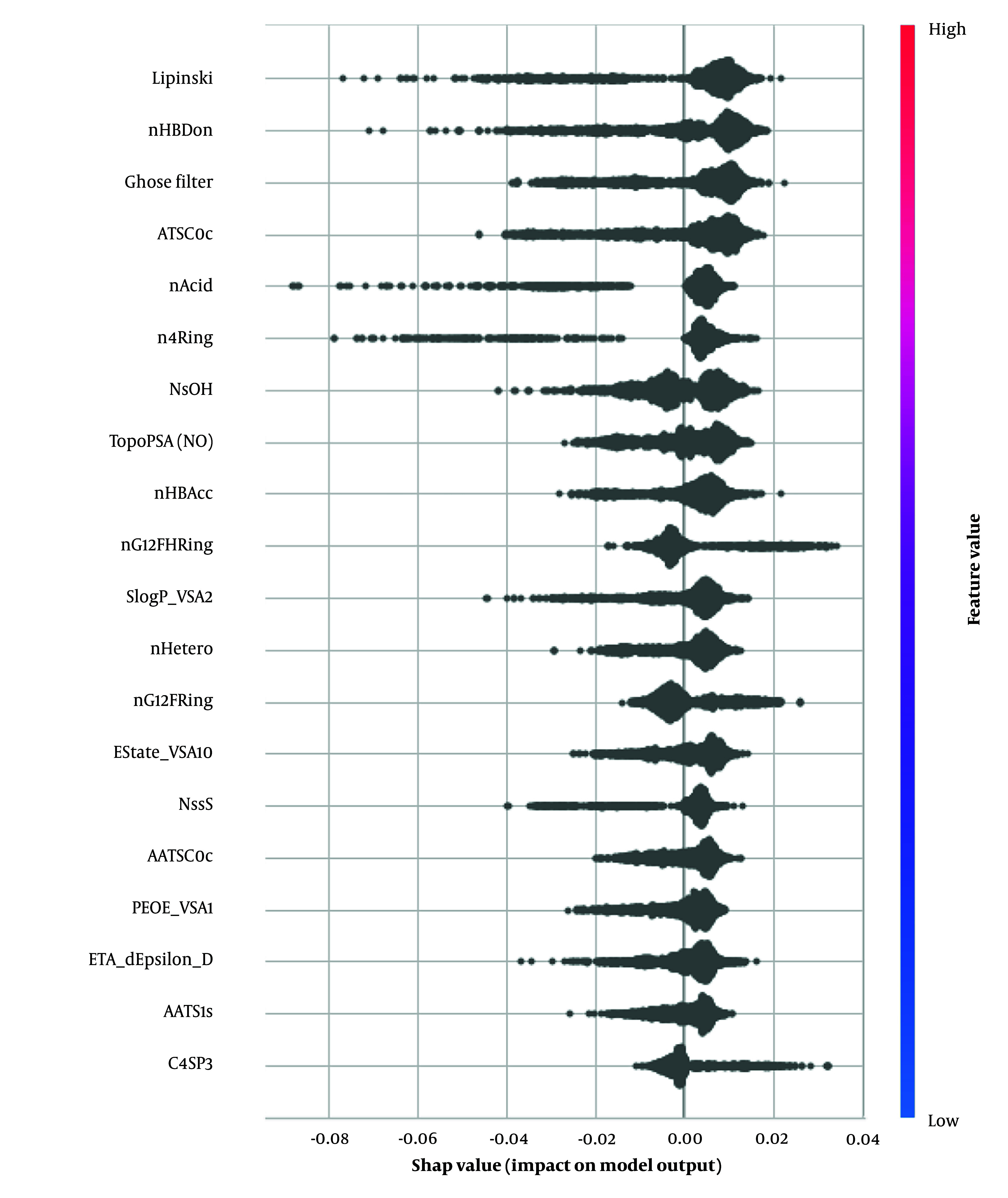
Summary plot of the distribution of importance for each feature overall molecule based on the results of extra trees classifier with Mordred chemical descriptors (MCDs)

We applied SHAP to our best-performing single model, the extra trees classifier. This model demonstrated higher performance metrics than all others, regardless of its combination with Morgan fingerprints or MCDs. The top five features identified as significant in predicting BBB permeability were: Lipinski rule of five (2D), number of hydrogen bond donors (nHBDon, 2D), Ghose filter (2D), centered Moreau-Broto autocorrelation of lag 0 weighted by Gasteiger charge (ATSC0c, 2D), and acidic group count (nAcid, 2D). A complete list of features is provided in Table 4 and Figure 5. 

**Table 4.** The First Ten Important Features in Permeability Prediction Based on Extra Trees Classifier with Mordred Chemical Descriptors Using Shapley Additive exPlanations Library

Abbreviation	Complete Name
**Lipinski**	Lipinski's rule of five
**nHBDon**	number of hydrogen bond donor
**GhoseFilter**	Ghose filter
**ATSC0c**	centered moreau-broto autocorrelation of lag 0 weighted by gasteiger charge
**nAcid**	acidic group count
**n4Ring**	membered ring count-4
**NsOH**	number of sOH
**TopoPSA (NO)**	topological polar surface area (use only nitrogen and oxygen)
**nHBAcc**	number of hydrogen bond acceptor
**nG12FHRing**	12-or-greater-membered fused hetero ring count

## 4. Discussion

### 4.1. Overview

In this study, we developed machine learning models to predict BBB permeability using the B3DB dataset, which includes data compiled from 50 credible sources. Four different versions of the dataset were prepared using the SMILES molecular representation for each drug, and the Pycaret library was utilized for training, validation, and testing of the models.

A significant challenge encountered was the imbalance in the dataset, which consisted of 4,956 positive instances and 2,851 negative ones. This imbalance made traditional evaluation metrics less reliable. To address this, we relied on the F1 Score and the MCC as our primary performance metrics, as they provide a more balanced assessment of model performance in imbalanced datasets.

### 4.2. Model Performance and Recommendation

Our research identified that the best-performing model was a voting ensemble model. This model, which we recommend as the optimal approach for similar datasets, integrated the extra trees classifier, Random Forest Classifier, and Gradient Boosting Classifier, all trained on MCDs. This ensemble model achieved outstanding results, with an AUC of 0.96, an F1 Score of 0.91, and an MCC of 0.74. The enhanced performance is likely attributable to the robust predictive capabilities of the MCDs and the complementary strengths of the classifiers within the ensemble.

The performance of our binary classification model was evaluated using the receiver operating characteristic (ROC) curve, which plots the true positive rate (TPR) against the false positive rate (FPR) at various classification thresholds. As shown in Figures 2 to 4, our models—extra trees classifier with ECFP6, Morgan Fingerprint, and MCDs—achieved AUC values of 0.93, 0.94, and 0.94, respectively, indicating excellent discriminative power.

The ROC curve was smooth and symmetrical across the entire region, suggesting consistent model performance across different thresholds. This high AUC value signifies that the model can reliably distinguish between positive and negative classes. The balance between TPR and FPR, as depicted by the ROC curve, indicates that the model maintains strong sensitivity and specificity across varying thresholds. This robust performance underscores the model's ability to generalize effectively to various scenarios.

### 4.3. Importance of Molecular Descriptors

We focused on the most significant molecular descriptors from the Mordred library, which have meaningful and verifiable implications in drug discovery. Utilizing the SHAP library, we identified the key descriptors influencing our model's outcomes. Lipinski's Rule of Five emerged as the most critical feature. This well-established guideline assesses whether a chemical compound with specific biological or pharmacological activity possesses properties that would make it likely to function as an orally active drug in humans. The rule suggests that an orally active drug should have no more than one violation of the following conditions: Molecular mass less than 500 Da, no more than five hydrogen bond donors, no more than ten hydrogen bond acceptors, and an octanol-water partition coefficient (log P) not greater than 5 (38).

Additionally, a variant of Lipinski's Rule for compounds targeting the CNS is Lipinski's Rule for CNS medications. This rule specifies a maximum of three hydrogen bond donors (HBDs), seven hydrogen bond acceptors (HBAs), a molecular weight (MW) under 400 Da, and a CLog P of at least five. Previous studies suggest that compounds adhering to this CNS-specific rule are more likely to penetrate the CNS effectively, thereby increasing their therapeutic potential (39).

As illustrated in Figure 5, key descriptors from the Mordred library were identified, with Lipinski's Rule of five emerging as the most critical. The plot demonstrates that compounds adhering to Lipinski's rules are more likely to be permeable through the BBB. The number of hydrogen bond donors (nHBDon) significantly impacted our findings, with most molecules showing positive SHAP values. This indicates that an increase in hydrogen bond donors generally enhances BBB permeability, which contrasts with the typical understanding that higher polarity reduces lipophilicity (40).

Interestingly, this finding diverges from a previous study by Yu et al., which combined machine learning and deep learning to develop a more interpretable approach to generalized rules for CNS drugs. Yu et al. found that the number of hydrogen bond donors was one of the essential features for classification, with a coefficient of -0.19. This negative coefficient indicates that an increase in hydrogen bonding reduces BBB penetration, aligning with the conventional understanding. Their model, which used a support vector machine (SVM) combined with a graph convolutional network, achieved an F1 score of 0.96 and an AUC of 0.97 on a dataset of 940 marketed drugs (41).

It is worth noting the discrepancy in the effect of the number of hydrogen bond donors observed in our study compared to Yu et al.'s findings. Another essential feature identified in our study was the number of acidic groups, which aligns with Yu et al.'s results. Their findings showed a coefficient of -0.16 for the number of acidic groups, indicating a similar impact on BBB permeability (41).

### 4.4. Comparative Analysis with Previous Studies

The size of a dataset can significantly influence the performance of machine learning models. Larger datasets often encompass more diversity and outliers, which can complicate the predictive task and make achieving higher accuracies more challenging. Moreover, imbalanced datasets can bias the model, resulting in poor predictive performance for the minority class (42). For example, in Saber et al.'s study, they utilized a dataset comprising 1,383 BBB+ and 310 BBB- cases, yielding a BBB+/BBB- ratio of 4.46, which is more imbalanced compared to our study's ratio of 1.74 (7,775 molecules). Despite this higher imbalance, Saber et al. achieved superior accuracy and F1 scores, with their SVM and QDA models achieving accuracies of 0.96, F1 scores of 0.98, and MCC scores of 0.88 and 0.87, respectively (12). In contrast, our study, which used a larger and more diverse dataset, resulted in a best-performing model (a voting ensemble model trained on MCDs) with an F1 score of 0.91 and an MCC of 0.74. This highlights the challenges introduced by greater dataset diversity.

To address the issue of imbalanced datasets, Wang et al. employed the Synthetic Minority Over-sampling Technique (SMOTE), which generates synthetic data to mitigate imbalance. Their study, which included 2,358 molecules (1,812 BBB+ and 546 BBB-), used various fingerprints and feature selection methods. Their most effective models, which combined SVM and KNN with SMOTE techniques, achieved an accuracy of 0.97, sensitivity of 0.99, specificity of 0.89, and an AUC of 0.919 (43). Despite their dataset's higher imbalance, their models demonstrated superior sensitivity but a lower AUC compared to our model. This underscores the importance of addressing data imbalance to achieve reliable predictive performance.

Another strategy for enhancing model performance without increasing dataset size is leveraging multiple data inputs. In Tang's 2022 study, they used a training set of 4,364 compounds (3,125 BBB+ and 1,239 BBB-) and a test set of 2,670 compounds (1,258 BBB+ and 1,412 BBB-). Their final dataset included 3,125 positive and 3,962 negative cases. Tang's study integrated three types of data inputs: Tabular data, SMILES text data, and chemical compound graphs. The model achieved an AUC of 0.83, sensitivity of 0.85, specificity of 0.64, accuracy of 0.74, and MCC of 0.49. The combination of text and tabular data yielded the best AUC of 0.82, with tabular data features such as 14-bit MACCS and 338-bit Morgan fingerprints emerging as the most significant (44). Although their study demonstrated lower accuracy and AUC compared to our model (accuracy of 0.88 and AUC of 0.96), it highlights the potential benefits of incorporating diverse input types. Exploring additional data inputs, such as text data and images, could further enhance model performance and represents a promising direction for future research.

### 4.5. Conclusions

In conclusion, our study demonstrated that the Light GBM model performed exceptionally well when using ECFP6 as input features, while the extra trees classifier excelled with Morgan fingerprints and MCDs. Our recommended ensemble model, which integrates the extra trees, random forest, and gradient boosting classifiers, achieved impressive results, including an AUC of 0.96, an F1 Score of 0.91, and an MCC of 0.74.

Key molecular descriptors identified in our analysis were Lipinski's rule of five (2D), the number of hydrogen bond donors (nHBDon, 2D), the Ghose filter (2D), centered Moreau-Broto autocorrelation of lag 0 weighted by Gasteiger charge (ATSC0c, 2D), and the acidic group count (nAcid, 2D). These findings align with previous studies that have established a correlation between Lipinski's Rule and the number of acidic groups with the permeability of CNS drugs, further validating the robustness and applicability of our models in predicting BBB permeability.

## Data Availability

The dataset presented in the study is available on request from the corresponding author during submission or after publication.
